# Treatment With Cytarabine at Initiation of Therapy With Cyclosporine and Glucocorticoids for Dogs With Meningoencephalomyelitis of Unknown Origin Is Not Associated With Improved Outcomes

**DOI:** 10.3389/fvets.2022.925774

**Published:** 2022-06-10

**Authors:** Renee Barber, Lauren Downey Koos

**Affiliations:** Department of Small Animal Medicine and Surgery, College of Veterinary Medicine, University of Georgia, Athens, GA, United States

**Keywords:** meningoencephalomyelitis of unknown origin (MUO), meningoencephalomyelitis of unknown etiology (MUE), meningoencephalitis, dog, cytosine arabinoside, cyclosporine

## Abstract

Meningoencephalomyelitis of unknown origin (MUO) is a common disorder of dogs that results in significant morbidity and mortality. The ideal treatment regimen is not known but a second immunosuppressive agent is often utilized in combination with glucocorticoids to increase efficacy and reduce side effects. Recently, a benefit to using a cytosine arabinoside (CA) constant rate infusion (CRI) at the time of diagnosis has been demonstrated. Here, a retrospective study was performed to determine if administration of CA at the time of diagnosis would alter prognosis in dogs receiving cyclosporine and prednisone for treatment of MUO. Medical records of 51 client-owned dogs diagnosed with MUO at one institution were reviewed (2009-2019). All dogs were treated with cyclosporine and a tapering course of prednisone. Twenty-one dogs received a single initial 200 mg/m^2^ treatment with CA either as a CRI or subcutaneously. Significantly more patients in the CA treatment group were obtunded on presentation but all other baseline parameters were similar between groups. No differences in success (defined as sustained improvement on neurological exam with owner perceived good quality of life), relapse, or death were identified at 1-, 3-, 6-, 9-, 12-, 18-, or 36-month time points. These results do not support treatment with CA (either as a CRI or subcutaneously) at the time of diagnosis in dogs treated with cyclosporine and prednisone.

## Introduction

Meningoencephalomyelitis of unknown origin (MUO) is a clinical diagnosis of non-infectious central nervous system (CNS) inflammation in dogs. It intended to represent a presumptive diagnosis of three histologically described variants of CNS inflammation that are overrepresented in young to middle-aged toy and small breed dogs: granulomatous meningoencephalomyelitis (GME), necrotizing meningoencephalitis (NME), and necrotizing leukoencephalitis (NLE). Although the etiopathogenesis of MUO has not been fully defined, an autoimmune etiology is suspected, and, as such, immunosuppression is the mainstay of treatment. To date, there is no consensus regarding a standard treatment protocol, and the prognosis for long-term survival remains guarded (1, 2).

Glucocorticoids are the cornerstone of treatment for MUO but a second immunosuppressive agent is often added in an attempt to increase efficacy and reduce steroid side effects. Cyclosporine is a commonly prescribed second agent that may prolong survival times when used in addition to glucocorticoids (3–6). However, cases treated with cyclosporine and glucocorticoids still have up to a 48% mortality rate with 78% of cases experiencing one or more relapse events (5).

Recent studies have shown that treatment with CA at the time of diagnosis can alter MUO prognosis. Dogs that received a constant rate infusion (CRI) of CA at the time of diagnosis had improved 3-month survival times compared to those that received subcutaneous CA (7). Additionally, patients receiving a single CRI of CA at the onset of treatment had the same prognosis as those that received a CRI of CA followed by subsequent, intermittent subcutaneous injections of CA over 72 weeks (8). This led to the hypothesis that addition of a single treatment with CA at the onset of diagnosis could improve outcomes in MUO-dogs treated with cyclosporine and prednisone.

In the retrospective study presented here, all dogs were treated with long-term cyclosporine and a tapering course of prednisone. One group received a single, additional treatment with CA at the time of diagnosis. Medical records were reviewed to determine outcomes at one, three, six, nine, 12, 18, and 36 months. The primary aim was to determine if short-term (i.e., 1 and 3-month) relapse and mortality rates were improved with addition of a single administration of cytarabine either intravenously or subcutaneously at the time of diagnosis. Additionally, based on the fact that mortality most commonly occurs within the 3 months following diagnosis of MUO (9, 10), a secondary hypothesis with that long-term (i.e., up to 36-month) relapse and mortality rates would also be improved with addition of CA.

## Materials and Methods

### Patient Selection

Medical records (2009–2019) were searched to identify dogs with a diagnosis of meningoencephalitis or meningoencephalomyelitis that also received cyclosporine and a tapering course of prednisone ± administration of a CA by CRI or subcutaneous injection at the time of treatment initiation.

Diagnostic inclusion criteria were based on a previous study with minor modifications (11): (i) availability of a complete medical record with neurological exams documented by either a neurology resident or board-certified neurologist; (ii) patient weight <15 kg; (iii) patient age 6 months to 12 years; (iv) focal or multifocal neuroanatomical lesion localization; (v) focal or multifocal T2-weighted hyperintensities on magnetic resonance imaging (MRI) consistent with inflammation; and (vi) cerebrospinal fluid (CSF) pleocytosis (greater than five total nucleated cells per microliter with <4,000 red blood cells per microliter) with >50% mononuclear cells. If necropsy confirmation of an MUO diagnosis was not available, dogs with a normal MRI, normal CSF, or from which CSF could not be obtained were excluded. Where possible, infectious diseases were ruled-out by negative serology for *Toxoplasma gondii, Neospora caninum*, and *Cryptococcus spp*. If serology for infectious diseases was not obtained, patients had to have a successful outcome (no relapse or death) for a minimum of 12 months while receiving immunosuppressive therapy or confirmation of GME, NME, or NLE by histopathology. Cases consistent with ischemic myelopathy (per-acute or acute history with clinical progression <48 h and focal T2W spinal cord hyperintensity without evidence of intracranial disease on exam or MRI) were excluded.

Additional criteria included treatment with cyclosporine (5–12 mg/kg/day) and prednisone (1.0–1.5 mg/kg/day) initiated at the time of diagnosis. Prednisone administration followed a routine tapering schedule of 25% every 4 weeks and was discontinued after every other day administration of 0.2–0.4 mg/kg/day for 4 weeks. Dogs in the CA treatment group had to receive 200 mg/m^2^ CA either as a CRI over eight to 12 h or divided into four subcutaneous injections given every 12 h. Dogs could not receive additional immunomodulatory therapy unless it was added after diagnosis of a relapse event. A minimum of 36 months follow-up was required.

### Data Collection

The following data were collected from each medical record: (i) signalment; (ii) time to presentation; (iii) history of seizures; (iv) presence or absence of obtundation; (v) complete neurological exams at 1, 3, 6, 9, 12, 18, and 36 months; (vi) MRI features including presence or absence of mass effect, sulci effacement, and transtentorial or foramen magnum herniation; (vii) CSF and infectious disease testing results; (viii) drug dosages, route of administration, and tapering protocol; (ix) survival times; and (x) necropsy results. Neurodisability scores (9) were calculated from recorded neurological exams.

### Treatment Groups

Patients were divided into two groups based on whether or not they received CA at the time of diagnosis. The CA+ group received CA 200 mg/m^2^ either as an 8 to 12-h CRI or four subcutaneous injections every 12 h. The CA- group did not receive any CA at the time of diagnosis, although it may have been subsequently administered at the time of a relapse.

### Outcome Assessment

At 1, 3, 6, n9, 12, 18, and 36 months, the following outcomes were determined and recorded: success, relapse, or death. Outcome success was defined as sustained neurological improvement (as determined by repeat neurological exams and concurrent owner report of satisfaction) after initiation of the treatment protocol. Diagnosis of relapse was based on recurrence of clinical signs that necessitated a change in treatment protocol, repeat MRI and / or CSF (where available), and expected response to alterations in treatment. Death was defined by all dogs that died or were euthanized due to clinical signs or complications related to the diagnosis of MUO. Relapse and death were only recorded once for each patient at the time of initial occurrence. Time to relapse, survival times, and change in neurodisability score were also determined as a secondary measure of outcome.

### Statistical Analysis

Baseline characteristics of dogs from each group were compared. Gender, history of seizures, presence or absence of obtundation, and MRI findings were compared using the Fisher exact test. Age, time to presentation, CSF total nucleated cell count (TNCC), CSF total protein, initial prednisone dose, and cyclosporine dose were compared using the Mann-Whitney *U*-test. Outcome measures of success and relapse were compared between groups using the chi-square test where all expected cell counts were greater than five. The outcome measure of death was compared between groups using Fisher exact test. Time to relapse and survival times were conducted using Kaplan-Meier plots and were compared between groups by log-rank analysis. Cases that died of causes not related to MUO were censored in the analysis. Statistical significance for all tests was initially set at *p* < 0.05. To account for the 21 simultaneous independent analyses performed for outcomes measurements (success, relapse and death), Bonferroni correction was applied resulting in a significance level of 0.0024.

## Results

### Patients

There were 21 dogs included in the CA+ group. Represented breeds included eight Maltese, three Chihuahuas, two Boston terriers, two French bulldogs, two miniature pinchers, two mixed breeds, and one each of rat terrier and Jack Russell terrier. There were 29 dogs included in the CA- group. Represented breeds included six Chihuahuas, five mixed breeds, three dachshunds, two French bulldogs, two Jack Russell terriers, two Maltese, two Yorkshire terriers, one affenpinscher, one American shepherd, one Boston terrier, one cairn terrier, one Pomeranian, one shi tzu, one silky terrier, and one toy poodle. There was no significant difference in gender, age at presentation, time to presentation, presence or absence of seizures, neurodisability score, presence of mass effect on MRI, evidence of increased intracranial pressure on MRI, or CSF results. There were significantly more obtunded dogs in CA+ group (43%) vs. the CA- group (13%) (*p* = 0.02) (Table 1). There were no significant differences in cyclosporine or prednisone doses between groups (Table 2). Prednisone was discontinued an average of 6 months after diagnosis in both groups. Cyclosporine was discontinued in two dogs after 12 months (both of which had successful outcomes), while all other dogs continued to receive cyclosporine for the 36-month study period. For the CA+ group, CA was administered *via* CRI in 12/21 patients (57%) and subcutaneously in 9/21 (43%). Necropsy results were available for three dogs and confirmed a diagnosis of GME in two dogs and NME in one dog.

**Table 1:** Patient variable at initial presentation.

	**CA+**	**CA–**	***p*-value**
Number of dogs	21	30	
**Gender**			
Male neutered	6	7	0.75
Female spayed	14	23	0.52
Female	1	0	0.41
Age at diagnosis (months)	56.5 (12–123)	67.5 (12–108)	0.33
Time to presentation (days)	19.5 (2–120)	15.2 (1–60)	0.83
**History/exam findings**			
Documentation of seizures	1/21 (5%)	4/30 (13%)	0.64
Patient obtunded	9/21 (43%)	4/30 (13%)	0.02
Neurodisability score	3.7 (1–7)	3.4 (1–7)	0.93
**MRI findings**			
Presence of mass effect	4/21 (19%)	3/30 (10%)	0.42
Presence of sulci effacement	1/21 (5%)	0/30 (0%)	0.41
Presence of transtentorial or foramen	1/21 (5%)	0/30 (0%)	0.41
magnum herniation			
CSF TNCC (cells/μl)	530 (6–2,271)	293 (6–1,933)	0.15
CSF total protein (mg/dl)	124 (20–348)	89 (18–371)	0.12

*CA+, cytosine arabinoside treatment group; CA–, non-cytosine arabinoside treatment group; TNCC, total nucleated cell count*.

*Results were considered statistically significant for a p-value of < 0.05*.

**Table 2:** Treatment parameters for dogs in the cytosine arabinoside and non-cytosine arabinoside treatment groups.

	**CA+**	**CA–**	***p*-value**
**Cytosar 200 mg/m** ^**2**^			
Constant rate infusion	12/21 (57%)		
Subcutaneous 4 doses over 48 h	9/21 (43%)		
Initial prednisone dose (mg/kg/day)	1.3 (1.0–1.5)	1.1 (1.0–1.5)	0.13
Cyclosporine dose (mg/kg/day)	7.8 (5–11)	7.4 (5–10)	0.28

*CA+, cytosine arabinoside treatment group; CA–, non-cytosine arabinoside treatment group*.

*Results were considered statistically significant for a p-value of < 0.05*.

### Outcomes

**Figure 1:**
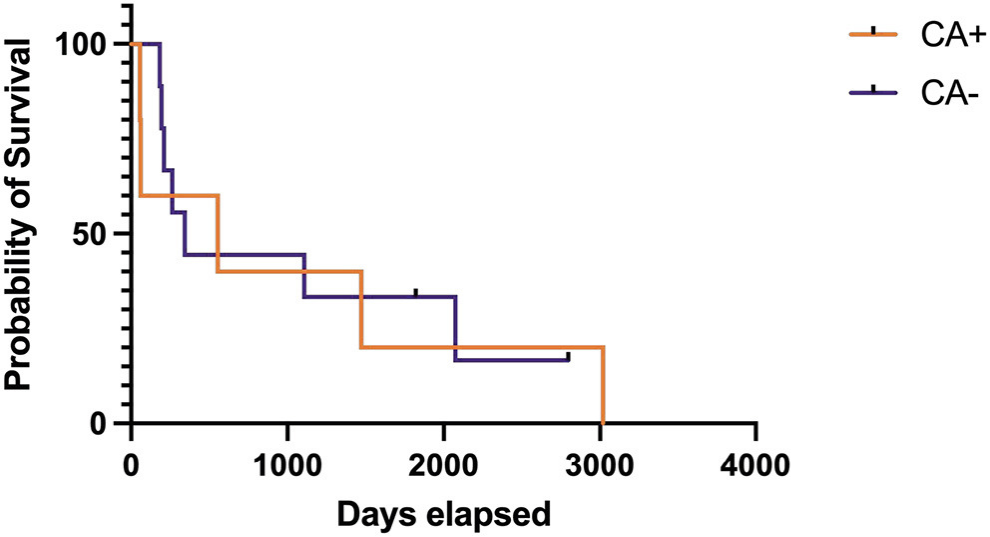
Kaplan-Meier survival care of deceased dogs with meningoencephalomyelitis of unknown origin treated with cyclosporine and prednisone with (CA+) or without (CA–) an initial dose of cytarabine (CA). Deaths not directly related to MUO or treatment of MUO have been censored and are noted by a vertical tick mark.

After Bonferroni correction, no statistically significant differences were identified between treatment groups for success, relapse, or death at any time point (Table 3). The median time to first relapse was 109 days (mean 284 days, range 89–864 days) for the CA+ group and 92 days (mean 181 days, range 24–732 days) for the CA- group (*p* = 0.35). Overall median survival times could not be calculated for the treatment groups as 16/21 dogs (76%) from the CA+ group and 24/30 dogs (80%) from the CA– group were alive or lost to follow-up at the time of writing; however, the median survival of those dogs had to exceed the follow-up time of 1,095 days. A Kaplan-Meier survival curve was generated for 5/21 dogs in the CA+ and 10/30 dogs in the CA- group that were followed until their death. For these deceased dogs, the median survival for the CA+ group was 555 days (range 57–3,018 days) and the median survival for the CA- group was 344 days (range 122–2,796 days) (*p* = 0.84) (Figure 1). For all dogs with a successful outcome (at any time point), neurodisability scores improved to <2 with residual deficits including one seizure less than every 2 months, head tilt, blindness, persistent lack of physiological nystagmus, and an ambulatory gait with mild weakness and / or ataxia.

**Table 3:** Success, relapse, and death rates for dogs in the cytosine arabinoside and non-cytosine arabinoside treatment groups.

	**CA+**	**CA–**	***p*-value**
**1 month**			
Success	19/21 (90%)	27/30 (90%)	0.95
Relapse	0/21 (0%)	3/30 (10%)	0.26
Death	2/21 (10%)	0/30 (0%)	0.16
**3 months**			
Success	16/21 (76%)	22/30 (73%)	0.82
Relapse	3/21 (14%)	8/30 (27%)	0.29
Death	2/21 (10%)	0/30 (0%)	0.16
**6 months**			
Success	15/21 (71%)	18/30 (60%)	0.40
Relapse	4/21 (19%)	10/30 (33%)	0.26
Death	2/21 (10%)	2/30 (7%)	1
**9 months**			
Success	15/21 (71%)	14/30 (47%)	0.08
Relapse	4/21 (19%)	11/30 (37%)	0.23
Death	2/21 (10%)	5/30 (16%)	0.68
**12 months**			
Success	15/21 (71%)	10/30 (33%)	0.007
Relapse	4/21 (19%)	14/30 (47%)	0.04
Death	2/21 (10%)	6/30 (20%)	0.44
**18 months**			
Success	13/21 (62%)	10/30 (33%)	0.04
Relapse	5/21 (24%)	14/30 (47%)	0.10
Death	3/21 (14%)	6/30 (20%)	0.72
**36 months**			
Success	12/21 (57%)	9/30 (30%)	0.05
Relapse	6/21 (29%)	15/30 (50%)	0.13
Death	3/21 (14%)	6/30 (20%)	0.72

*CA+, cytosine arabinoside treatment group; CA–, non-cytosine arabinoside treatment group*.

*Bonferroni correction was applied to account for multiple independent analysis. Results were considered statistically significant for a p-value of < 0.0024*.

## Discussion

We retrospectively evaluated supplemental use of CA as a single treatment at the time of diagnosis in dogs being treated for MUO with cyclosporine and prednisone. Although relapse and death rates were lower for the CA+ group at all time points, no statistically significant changes in outcome were identified. As such, the results of this study do not support addition of CA to a treatment protocol with cyclosporine and prednisone.

There are several study limitations that must be considered when interpreting these results. First, this is a retrospective study, which may have led to falsely elevated survival times. Patients were only included if they were started on a treatment regimen with cyclosporine and prednisone, which likely excluded some dogs that died prior to institution of oral medications. Additionally, although statistical significance was not reached at any time point in our study, investigation with larger case numbers might better elucidate differences in the treatment groups. Bonferroni correction was used to account for multiple simultaneous analyses. Although this conservative correction helps limit type I error, it can also lead to higher rates of false negatives. Other limitations include the fact that histological confirmation of diagnosis was only available in a minority of cases. Also, in the majority of dogs, diagnosis of relapse was based on recurrence of clinical signs and response to changes in therapy. Since numerous neurological conditions respond to treatment with prednisone, it is possible that some dogs with suspected relapse had a separate condition.

The findings in this study differ from other reports where majority of death occurs within the first 3 months after diagnosis (7, 9, 10). There were a total of nine deaths in this study but only two of these occurred before 3 months. The lower death rate early in the study might be accounted for by the previously mentioned limitation that cases were excluded if they died before administration of oral medications. But this does not account for the higher number of deaths after 3 months than previously reported. Looking more closely at the cases that died after 3 months, the majority were euthanatized associated with a severe relapse, often after tapering of prednisone to a dosage of <0.4 mg/kg/day. It should also be considered that cases included here received a lower initial prednisone dosage (maximum 1.5 mg/kg/day) than many other studies that administer 2.0 mg/kg/day. This justification for this lower dosage is anecdotal; the authors have noted fewer prednisone side effects and owner complaints with no appreciable difference in outcomes. Unfortunately, it is difficult to compare outcomes between manuscripts so a dedicated study to evaluate the effects of these lower prednisone dosages is required.

Another point to consider is how CA was administered in our study. Administration of CA as a CRI instead of subcutaneously at the time of diagnosis has been shown to improve 3-month survival times (7). Patients in our study received CA either as a CRI or subcutaneously, but statistical analysis to identify differences in these groups was not possible due to small case numbers. It is unclear if outcomes would have been improved if all dogs in this study had received a CRI instead of subcutaneous injections, but this should be considered when planning future studies.

For future studies, it is probably important to better qualify the impact of relapses on patient and owner quality of life. Multiple relapses were not addressed in this study but it must be acknowledged that not all relapses have the same impact. Many relapses are mild and respond to a minor medication adjustment like increasing the prednisone dose, while others are more severe or occur repeatedly and cause emotional and financial strain for owners.

In summary, we failed to identify improved outcomes in dogs with MUO that received a supplemental treatment of CA at the time of diagnosis in addition to treatment with cyclosporine and prednisone. Because there was a trend toward improved outcomes in the CA+ group that did not reach statistical significance, there may be benefit to a prospective, randomized, double-blinded treatment trial that could investigate similar treatment protocols in a larger number of cases.

## Data Availability Statement

The original contributions presented in the study are included in the article/supplementary material, further inquiries can be directed to the corresponding author.

## Author Contributions

RB was responsible for study design and data collection. RB and LD were responsible for manuscript preparation. All authors approved the submitted version of the manuscript.

## Conflict of Interest

The authors declare that the research was conducted in the absence of any commercial or financial relationships that could be construed as a potential conflict of interest. The handling editor AT, declared a past co-authorship with the author, RB.

## Publisher's Note

All claims expressed in this article are solely those of the authors and do not necessarily represent those of their affiliated organizations, or those of the publisher, the editors and the reviewers. Any product that may be evaluated in this article, or claim that may be made by its manufacturer, is not guaranteed or endorsed by the publisher.

## References

[1] Coates JR Jeffery ND. Perspectives on meningoencephalomyelitis of unknown origin. Vet Clin North Am Small Anim Pract. (2014) 44:1157–85. 10.1016/j.cvsm.2014.07.009 25239815

[2] Cornelis I Van Ham L Gielen I De Decker S Bhatti SFM. Clinical presentation, diagnostic findings, prognostic factors, treatment and outcome in dogs with meningoencephalomyelitis of unknown origin: a review. Vet J. (2019) 244:37–44. 10.1016/j.tvjl.2018.12.007 30825893

[3] Adamo PF Rylander H Adams WM. Ciclosporin use in multi-drug therapy for meningoencephalomyelitis of unknown aetiology in dogs. J Small Anim Pract. (2007) 48:486–96. 10.1111/j.1748-5827.2006.00303.x 17617166

[4] Jung DI Kang BT Park C Yoo JH Gu SH Jeon HW. A comparison of combination therapy (cyclosporine plus prednisolone) with sole prednisolone therapy in 7 dogs with necrotizing meningoencephalitis. J Vet Med Sci. (2007) 69:1303–6. 10.1292/jvms.69.1303 18176031

[5] Brady SL Woodward AP le Chevoir MAR. Survival time and relapse in dogs with meningoencephalomyelitis of unknown origin treated with prednisolone and ciclosporin: a retrospective study. Aust Vet J. (2020) 98:491–8. 10.1111/avj.12994 32794230

[6] Paušová TK Tomek A Šrenk P Belašková S. Clinical presentation, diagnostic findings, and long-term survival time in 182 dogs with meningoencephalitis of unknown origin from central Europe that were administered glucocorticosteroid monotherapy. Top Comp Anim Med. (2021) 44:100539. 10.1016/j.tcam.2021.100539 33964477

[7] Lowrie M Thomson S Smith P Garosi L. Effect of a constant rate infusion of cytosine arabinoside on mortality in dogs with meningoencephalitis of unknown origin. Vet J. (2016) 213:1–5. 10.1016/j.tvjl.2016.03.026 27240905

[8] Stee K Broeckx BJ Targett M Gomes SA Lowrie M. Cytosine arabinoside constant rate infusion without subsequent subcutaneous injections for the treatment of dogs with meningoencephalomyelitis of unknown origin. Vet Rec. (2020) 187:e98. 10.1136/vr.106019 32862133

[9] Smith PM Stalin CE Shaw D Granger N Jeffery ND. Comparison of two regimens for the treatment of meningoencephalomyelitis of unknown etiology. J Vet Int Med. (2009) 23:520–6. 10.1111/j.1939-1676.2009.0299.x 19645837

[10] Lowrie M Smith PM Garosi L. Meningoencephalitis of unknown origin: investigation of prognostic factors and outcome using a standard treatment protocol. Vet Rec. (2013) 172:527–527. 10.1136/vr.101431 23462382

[11] Granger N Smith PM Jeffery ND. Clinical findings and treatment of non-infectious meningoencephalomyelitis in dogs: a systematic review of 457 published cases from 1962 to 2008. Vet J. (2010) 184:290–7. 10.1016/j.tvjl.2009.03.031 19410487

